# Supplementary material for: The Evolution and Characterization of the RNA Interference Pathways in Lophotrochozoa
Source: Genome Biol Evol. 2024 May 7;16(5):evae098. doi: 10.1093/gbe/evae098 (PMC11114477; doi:10.1093/gbe/evae098)
Supplement: evae098_Supplementary_Data [file evae098_supplementary_data.zip › Supplementary_materials.zip!/selected_miRNAs/NoGe_selected_miRNAs/mirdeep_pdf_files/NMRB01001213.1_Notospermus_geniculatus_strain_Ushimado_scaffold1213,_whole_genome_shotgun_sequence_26243.pdf]

Provisional ID	:	NMRB01001213.1_Notospermus_geniculatus_strain_Ushimado_scaffold1213,_whole_genome_shotgun_sequence_26243
Score total	:	211241.2
Score for star read(s)	:	3.9
Score for read counts	:	211232.4
Score for mfe	:	1.8
Score for randfold	:	0
Score for cons. seed	:	3
Total read count	:	414335
Mature read count	:	413431
Loop read count	:	0
Star read count	:	904

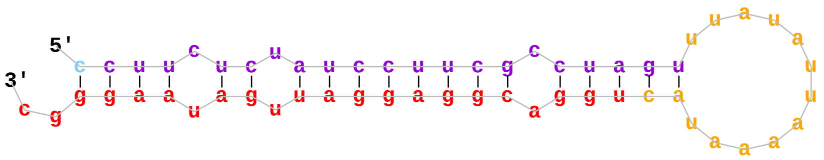

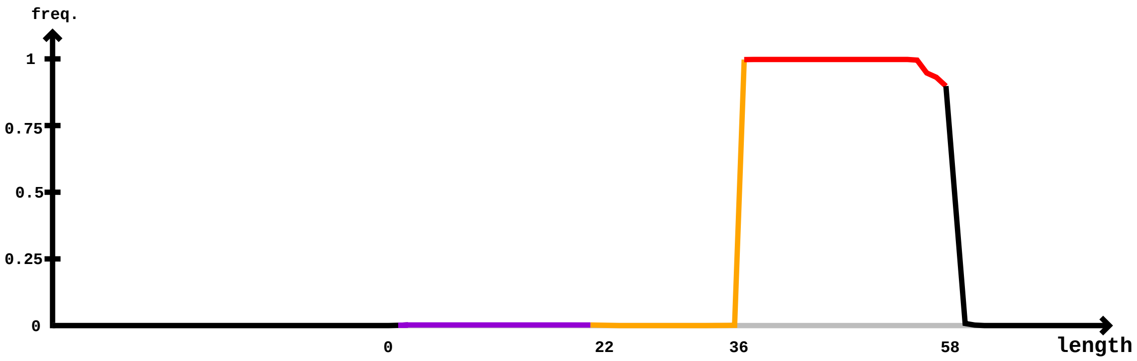


```
                                      Star                              Mature
5'- guguagugaagacugaagguaguucuuagcgaguccuucucuauccuucgccuaguuuauauuaaaaauacuggacggaggauugauaagggcuugcggggacacgguc  -3'  obs
    guguagugaagacugaagguaguucuuagcgaguccuucucuauccuucgccuaguuuauauuaaaaauacuggacggaggauugauaagggcuugcggggacacgguc       exp
    ...((((....))))...((.((((((.((((((((((.((.((((((((.(((((.............))))).)))))))).)).))))))))))))))))))....   reads   mm   sample
    ..................................ccuucucuauccuucgccC........................................................   1       1    Fm1
    ..................................ccuucucuauccuucgccu........................................................   5       0    Fm1
    ..................................ccuucucuauccuucgccuagu.....................................................   67      0    Fm1
    ..................................ccuucucuauccuucgcAuagu.....................................................   1       1    Fm1
    ..................................ccuucCcuauccuucgccuagu.....................................................   1       1    Fm1
    ..................................ccuucucuauccuucgccuaguA....................................................   2       1    Fm1
    ...................................cuucucuauccuucgccu........................................................   1       0    Fm1
    ...................................cuucucCauccuucgccu........................................................   1       1    Fm1
    ...................................cuucucuauccuucgccuag......................................................   4       0    Fm1
    ...................................cuucucuauUcuucgccuagu.....................................................   3       1    Fm1
    ...................................cuucucuauccuucgccuagu.....................................................   188     0    Fm1
    ...................................cuucucuaucAuucgccuagu.....................................................   1       1    Fm1
    ...................................cuuUucuauccuucgccuagu.....................................................   1       1    Fm1
    ...................................cuucucuauccuucgccCagu.....................................................   2       1    Fm1
    ...................................cuucucuauccuucgcAuagu.....................................................   1       1    Fm1
    ...................................cuucucuauccuucgccuagG.....................................................   1       1    Fm1
    ...................................cuucucuauccuucgccGagu.....................................................   2       1    Fm1
    ...................................cuucAcuauccuucgccuagu.....................................................   1       1    Fm1
    ...................................cuucucuauccuucgccuaguu....................................................   2       0    Fm1
    ...................................cuucucuauccuucgccuaguA....................................................   5       1    Fm1
    .....................................................................acuggacggaggauugauaagg..................   5       0    Fm1
    .....................................................................acuggacggaggauugauaagggc................   2       0    Fm1
    .....................................................................Ucuggacggaggauugauaagggc................   1       1    Fm1
    ......................................................................cuggacggaggaCugauaa....................   1       1    Fm1
    ......................................................................cuggacggaggauugauaag...................   6       0    Fm1
    ......................................................................cuggacggaggauugauaagggA................   1       1    Fm1
    ......................................................................cuggacggaggauugauaagAgc................   1       1    Fm1
    ......................................................................cuggacggGggauugauaagggc................   1       1    Fm1
    ......................................................................cuggacggaggaCugauaagggc................   4       1    Fm1
    ......................................................................cuggacggaggauugauaagggc................   32      0    Fm1
    ......................................................................cuggacggagAauugauaagggc................   1       1    Fm1
    .......................................................................uggacggaggauugaAaa....................   6       1    Fm1
    .......................................................................uggacggaggauugauaa....................   63      0    Fm1
```

Star Mature

```
guguagugaagacugaagguaguucuuagcgaguccuucucuauccuucgccuaguuuauauuaaaauacuggacggaggauugauaagggcuugcggggacacgguc
......................................................................uggacggaggauugauUa....................   1      1   Fm1
......................................................................Nggacggaggauugauaag...................   3      1   Fm1
......................................................................uggacggaggauugauaaC...................   1      1   Fm1
......................................................................uggacggaggUuugauaag...................   6      1   Fm1
......................................................................uggacggaggauugauCag...................   4      1   Fm1
......................................................................uggacggaggCuugauaag...................   2      1   Fm1
......................................................................uggacggagAauugauaag...................   8      1   Fm1
......................................................................uggacggaggauugauaaA...................   128    1   Fm1
......................................................................uggacggaggauuUauaag...................   2      1   Fm1
......................................................................uggacggaggaCugauaag...................   9      1   Fm1
......................................................................ugAacggaggauugauaag...................   1      1   Fm1
......................................................................uggacggaUgauugauaag...................   1      1   Fm1
......................................................................uggacggaggauugauaaU...................   50     1   Fm1
......................................................................uggacggaggauugauaUg...................   9      1   Fm1
......................................................................uggacggaggauugauaCg...................   4      1   Fm1
......................................................................uggacggaggGuugauaag...................   1      1   Fm1
......................................................................uggacggaggauAgauaag...................   1      1   Fm1
......................................................................uggaUggaggauugauaag...................   1      1   Fm1
......................................................................uggacggaggauuAauaag...................   1      1   Fm1
......................................................................uggacggaggauugauGag...................   2      1   Fm1
......................................................................uggacggaggauugauaag...................   1483   0   Fm1
......................................................................uggacggaggGuugauaagg..................   1      1   Fm1
......................................................................uAgacggaggauugauaagg..................   1      1   Fm1
......................................................................uggacggagAauugauaagg..................   8      1   Fm1
......................................................................uggacggUggauugauaagg..................   2      1   Fm1
......................................................................uggacggaggCuugauaagg..................   2      1   Fm1
......................................................................uggacggaggauugauaagg..................   670    0   Fm1
......................................................................uggacggaggUuugauaagg..................   3      1   Fm1
......................................................................uggacggaggauugauUagg..................   1      1   Fm1
......................................................................uggacggaggaCugauaagg..................   51     1   Fm1
......................................................................uggacggaggauugaCaagg..................   1      1   Fm1
......................................................................uggacggaggauugauaagU..................   15     1   Fm1
......................................................................uggacggaggauugauaagA..................   74     1   Fm1
......................................................................uggacggaggauugauCagg..................   1      1   Fm1
......................................................................uggacggaggauuCauaagg..................   1      1   Fm1
......................................................................uggacggaggauugUuaaggg.................   1      1   Fm1
......................................................................uggacggagAauugauaaggg.................   20     1   Fm1
......................................................................ugUacggaggauugauaaggg.................   1      1   Fm1
......................................................................uggacggaggauugauaaggg.................   1456   0   Fm1
......................................................................uggacAgaggauugauaaggg.................   1      1   Fm1
......................................................................uggCcggaggauugauaaggg.................   1      1   Fm1
......................................................................uggacggaggauugauaaggC.................   9      1   Fm1
......................................................................ugCacggaggauugauaaggg.................   1      1   Fm1
......................................................................uggacgAaggauugauaaggg.................   1      1   Fm1
......................................................................uggacggaggGuugauaaggg.................   3      1   Fm1
......................................................................uggacggaggauugauCaggg.................   6      1   Fm1
......................................................................uggacggaggaCugauaaggg.................   59     1   Fm1
......................................................................uggacggaggUuugauaaggg.................   1      1   Fm1
......................................................................uggaGggaggauugauaaggg.................   1      1   Fm1
......................................................................uggacggaggauuUauaaggg.................   1      1   Fm1
......................................................................uggaUggaggauugauaaggg.................   1      1   Fm1
......................................................................uggacggaCgauugauaaggg.................   1      1   Fm1
......................................................................uggacggaggauugauaGggg.................   11     1   Fm1
......................................................................uUgacggaggauugauaaggg.................   1      1   Fm1
......................................................................uggacggaggauugauaaggU.................   15     1   Fm1
......................................................................uggacggaggauugauaCggg.................   5      1   Fm1
......................................................................uggacggGggauugauaaggg.................   1      1   Fm1
......................................................................uggGcggaggauugauaaggg.................   1      1   Fm1
......................................................................Nggacggaggauugauaaggg.................   2      1   Fm1
......................................................................uggacggaggauugauaaggA.................   153    1   Fm1
......................................................................uggacgUaggauugauaaggg.................   1      1   Fm1
......................................................................uggacggaggauugauaUggg.................   4      1   Fm1
......................................................................uggacggaggauugauGaggg.................   2      1   Fm1
......................................................................uggacggaggaAugauaaggg.................   1      1   Fm1
......................................................................uggacggaggauugauUaggg.................   6      1   Fm1
......................................................................uAgacggaggauugauaaggg.................   2      1   Fm1
......................................................................uggacggaggauugGuaagggc................   8      1   Fm1
......................................................................uggacAgaggauugauaagggc................   8      1   Fm1
......................................................................uggacggaggaAugauaagggc................   9      1   Fm1
......................................................................uggacCgaggauugauaagggc................   2      1   Fm1
```

```
                                    Star                                Mature
guguagugaagacugaagguaguucuuagcgagucCUUCUCUAUCCUUCGCCUAGUuuauauuaaaauacuggacggaggauugauaagggcuugcggggacacgguc
......................................................................uggacggaggauuCauaagggc................ 3      1  Fm1
......................................................................uggacggaggauugauaagggA................ 652    1  Fm1
......................................................................uggacggaggauugauaagCgc................ 5      1  Fm1
......................................................................ugNacggaggauugauaagggc................ 1      1  Fm1
......................................................................uggaUggaggauugauaagggc................ 22     1  Fm1
......................................................................uggacggagUauugauaagggc................ 14     1  Fm1
......................................................................uggacggCggauugauaagggc................ 15     1  Fm1
......................................................................uggacggaggauugauaagggG................ 13     1  Fm1
......................................................................uggacggaggauugauaagUgc................ 21     1  Fm1
......................................................................uNgacggaggauugauaagggc................ 1      1  Fm1
......................................................................uggacggaggauuAauaagggc................ 10     1  Fm1
......................................................................uggacggaggauugauaaAggc................ 7      1  Fm1
......................................................................uggacgNaggauugauaagggc................ 6      1  Fm1
......................................................................ugUacggaggauugauaagggc................ 13     1  Fm1
......................................................................ugAacggaggauugauaagggc................ 16     1  Fm1
......................................................................uggacggaAgauugauaagggc................ 4      1  Fm1
......................................................................uggacggaggauGgauaagggc................ 2      1  Fm1
......................................................................uggacggaggauugauaaggAc................ 14     1  Fm1
......................................................................uggacggaggaGugauaagggc................ 6      1  Fm1
......................................................................uggacggaggauugauaaggUc................ 19     1  Fm1
......................................................................uCgacggaggauugauaagggc................ 11     1  Fm1
......................................................................uggaGggaggauugauaagggc................ 8      1  Fm1
......................................................................uggacggaggauCgauaagggc................ 13     1  Fm1
......................................................................uggacggUggauugauaagggc................ 66     1  Fm1
......................................................................uggacggaggauugaCaagggc................ 5      1  Fm1
......................................................................uggacggagAauugauaagggc................ 284    1  Fm1
......................................................................ugCacggaggauugauaagggc................ 6      1  Fm1
......................................................................uggacggaggauugauaCgggc................ 281    1  Fm1
......................................................................uggacggaggauuUauaagggc................ 4      1  Fm1
......................................................................uggacggaggauugauUagggc................ 110    1  Fm1
......................................................................Nggacggaggauugauaagggc................ 25     1  Fm1
......................................................................uggacggaggauugUuaagggc................ 19     1  Fm1
......................................................................uggacggaggUuugauaagggc................ 173    1  Fm1
......................................................................uggacggaCgauugauaagggc................ 2      1  Fm1
......................................................................Cggacggaggauugauaagggc................ 10     1  Fm1
......................................................................uggacggaggauugauaaggCc................ 5      1  Fm1
......................................................................uggacggaggauAgauaagggc................ 5      1  Fm1
......................................................................uggacggaggaCugauaagggc................ 1684   1  Fm1
......................................................................uggacggagCauugauaagggc................ 5      1  Fm1
......................................................................uggacggaggCuugauaagggc................ 107    1  Fm1
......................................................................uggacggaggauugauaGgggc................ 236    1  Fm1
......................................................................uggacggaggauugauaaCggc................ 5      1  Fm1
......................................................................uUgacggaggauugauaagggc................ 4      1  Fm1
......................................................................uggacggaggauugauaaUggc................ 10     1  Fm1
......................................................................uggacggGggauugauaagggc................ 92     1  Fm1
......................................................................uggacggaggauugCuaagggc................ 5      1  Fm1
......................................................................uggacggaggauugauaUgggc................ 246    1  Fm1
......................................................................uAgacggaggauugauaagggc................ 36     1  Fm1
......................................................................uggacggaggGuugauaagggc................ 107    1  Fm1
......................................................................uggacggaggauugauaagggU................ 193    1  Fm1
......................................................................uggacggaggauugauaagAgc................ 15     1  Fm1
......................................................................uggacggaggauugauGagggc................ 88     1  Fm1
......................................................................uggacNgaggauugauaagggc................ 13     1  Fm1
......................................................................uggaAggaggauugauaagggc................ 6      1  Fm1
......................................................................uggacgUaggauugauaagggc................ 12     1  Fm1
......................................................................uggacggaggauugauCagggc................ 154    1  Fm1
......................................................................uggGcggaggauugauaagggc................ 10     1  Fm1
......................................................................uggacgAaggauugauaagggc................ 6      1  Fm1
......................................................................uggCcggaggauugauaagggc................ 11     1  Fm1
......................................................................uggUcggaggauugauaagggc................ 16     1  Fm1
......................................................................uggacUgaggauugauaagggc................ 10     1  Fm1
......................................................................uggacgCaggauugauaagggc................ 4      1  Fm1
......................................................................Aggacggaggauugauaagggc................ 3      1  Fm1
......................................................................uggacggaUgauugauaagggc................ 11     1  Fm1
......................................................................uggacggaggauugauaagggc................ 73857  0  Fm1
......................................................................uggacggagAauugauaagggcu............... 12     1  Fm1
......................................................................uggacgAaggauugauaagggcu............... 2      1  Fm1
......................................................................uggacggaggauugauaagggGu............... 1      1  Fm1
......................................................................uggacggaggauugauGagggcu............... 6      1  Fm1
......................................................................uggaAggaggauugauaagggcu............... 1      1  Fm1
```

```
                                   Star                              Mature
guguagugaagacugaagguaguucuuagcgaguccuucucuauccuucgccuaguuuauauuaaaauacuggacggaggauugauaagggcuugcggggacacgguc
......................................................................uggacggaggauugauaagggcC...............  15    1  Fm1
......................................................................uggacggaggauugauaaggUcu...............  1     1  Fm1
......................................................................uggacggGggauugauaagggcu...............  2     1  Fm1
......................................................................uggacggaggauugauaagggAu...............  8     1  Fm1
......................................................................uggacggaggCuugauaagggcu...............  12    1  Fm1
......................................................................Cggacggaggauugauaagggcu...............  1     1  Fm1
......................................................................uAgacggaggauugauaagggcu...............  3     1  Fm1
......................................................................uggacCgaggauugauaagggcu...............  1     1  Fm1
......................................................................uggacggaggauugauaUgggcu...............  15    1  Fm1
......................................................................uggacgNaggauugauaagggcu...............  1     1  Fm1
......................................................................uggacggaCgauugauaagggcu...............  1     1  Fm1
......................................................................uggacggaggauuAauaagggcu...............  1     1  Fm1
......................................................................uggacggaggauugauaaUggcu...............  1     1  Fm1
......................................................................uggacggaggauugaAaagggcu...............  1     1  Fm1
......................................................................uggacggaggauugCuaagggcu...............  1     1  Fm1
......................................................................uggacggUggauugauaagggcu...............  7     1  Fm1
......................................................................uggacggaggauugauUagggcu...............  9     1  Fm1
......................................................................uggaGggaggauugauaagggcu...............  1     1  Fm1
......................................................................uggacggaggGuugauaagggcu...............  12    1  Fm1
......................................................................uggacggaAgauugauaagggcu...............  1     1  Fm1
......................................................................uggacggaggaAugauaagggcu...............  2     1  Fm1
......................................................................uggaUggaggauugauaagggcu...............  1     1  Fm1
......................................................................uggGcggaggauugauaagggcu...............  1     1  Fm1
......................................................................uggacggagUauugauaagggcu...............  1     1  Fm1
......................................................................uggacggaggauugauCagggcu...............  17    1  Fm1
......................................................................uggacggaggauugauaGgggcu...............  19    1  Fm1
......................................................................ugUacggaggauugauaagggcu...............  2     1  Fm1
......................................................................uggacggaggauuCauaagggcu...............  1     1  Fm1
......................................................................uggacggaggauugauaagggcu...............  4960  0  Fm1
......................................................................uggacggaggauugauaCgggcu...............  14    1  Fm1
......................................................................uggacggaggauugGuaagggcu...............  1     1  Fm1
......................................................................uggacggaggauugauaagggcG...............  150   1  Fm1
......................................................................uggacggaggauugauaagggcA...............  1002  1  Fm1
......................................................................uggacggaggUuugauaagggcu...............  13    1  Fm1
......................................................................Nggacggaggauugauaagggcu...............  2     1  Fm1
......................................................................uggacggaggauugauaagggUu...............  17    1  Fm1
......................................................................uggacggaggaCugauaagggcu...............  74    1  Fm1
......................................................................uggacggaggauugauaagggcAu..............  124   1  Fm1
......................................................................uggacggaggauugaCaagggcuu..............  1     1  Fm1
......................................................................uggacggaggCuugauaagggcuu..............  1     1  Fm1
......................................................................uggacggCggauugauaagggcuu..............  1     1  Fm1
......................................................................uggacggUggauugauaagggcuu..............  1     1  Fm1
......................................................................uggacggaggauugUuaagggcuu..............  1     1  Fm1
......................................................................uggacggaggauugauaCgggcuu..............  1     1  Fm1
......................................................................uggacggaggauugauaagggUuu..............  5     1  Fm1
......................................................................uggacggaggauugauUagggcuu..............  1     1  Fm1
......................................................................uggacggaggaCugauaagggcuu..............  10    1  Fm1
......................................................................uggacggagAauugauaagggcuu..............  12    1  Fm1
......................................................................Nggacggaggauugauaagggcuu..............  1     1  Fm1
......................................................................uggacggaggauugauaagggAuu..............  7     1  Fm1
......................................................................uggacggaggauugauaUgggcuu..............  1     1  Fm1
......................................................................uggacggaggauugauaagggcuG..............  10    1  Fm1
......................................................................uggacggaggauugauaagggcuA..............  16    1  Fm1
......................................................................uggacggaggUuugauaagggcuu..............  3     1  Fm1
......................................................................uggacggaggauugauaagggcCu..............  4     1  Fm1
......................................................................uggUcggaggauugauaagggcuu..............  1     1  Fm1
......................................................................uggacggaggauugauaagggcuu..............  671   0  Fm1
......................................................................uggacggaggauugauCagggcuu..............  1     1  Fm1
......................................................................uggacggaggGuugauaagggcuu..............  1     1  Fm1
......................................................................uggacggaggauugauaagggcuuU.............  334   1  Fm1
......................................................................uggacggaggauugauaagggcuug.............  2     0  Fm1
......................................................................uggacggaggauugauaagggcuuA.............  3     1  Fm1
.......................................................................ggacggaggaCugauaag...................  1     1  Fm1
.......................................................................ggacggaggauugauaag...................  1     0  Fm1
.......................................................................ggacggaggauugauaagg..................  1     0  Fm1
.......................................................................ggacggaggaGugauaaggg.................  1     1  Fm1
.......................................................................ggacggagAauugauaagggc................  4     1  Fm1
.......................................................................ggacggagCauugauaagggc................  1     1  Fm1
.......................................................................ggacggaggauugauaagggc................  7     0  Fm1
.......................................................................ggacggaggaCugauaagggc................  4     1  Fm1
```

Star **Mature**

```
guguagugaagacugaagguaguucuuagcgaguccuucucuauccuucgccuaguuuauauuaaaaauacuggacggaggauugauaagggcuugcggggacacgguc
........................................................................Ugacggaggauugauaagggc................   4     1     Fm1
........................................................................ggacggaggauugauaagggcA...............   3     1     Fm1
........................................................................Ugacggaggauugauaagggcu...............   1     1     Fm1
........................................................................ggacggaggauugauaagggcu...............   1     0     Fm1
..........................................................................acggaggauugauaagggc................   7     0     Fm1

..................................ccuucucuauccuucgccuagu.....................................................   21    0     Fm2
..................................ccuucucuauGcuucgccuagu.....................................................   1     1     Fm2
..................................ccuucucuauccuucgAcuagu.....................................................   1     1     Fm2
...................................cuucucuauccuucgccGagu.....................................................   1     1     Fm2
...................................cuucucuauccuucgccuagu.....................................................   46    0     Fm2
...................................cuucucuauccuucgcAuagu.....................................................   1     1     Fm2
...................................cuucucuauccuucgccuaguu....................................................   7     0     Fm2
....................................................................uacuggacggaggauugauaa....................   5     0     Fm2
....................................................................uacuggacggaggauugGuaa....................   1     1     Fm2
......................................................................cuggacggaggaCugauaagg..................   1     1     Fm2
......................................................................cuggacggagAauugauaagggc................   4     1     Fm2
......................................................................cuggacggaggauugauaagggc................   4     0     Fm2
......................................................................cuggacggaggauugauaagggcu...............   6     0     Fm2
.......................................................................uggacggaggauugaAaa....................   3     1     Fm2
.......................................................................uggacggaggauugauaa....................   24    0     Fm2
.......................................................................uggacggaggauugauaCg...................   3     1     Fm2
.......................................................................uggaUggaggauugauaag...................   1     1     Fm2
.......................................................................uggacggaggauugauaUg...................   1     1     Fm2
.......................................................................uggacggagAauugauaag...................   2     1     Fm2
.......................................................................uggacggaggaCugauaag...................   9     1     Fm2
.......................................................................uggacggaggGuugauaag...................   2     1     Fm2
.......................................................................uggacggaggauugauaaU...................   36    1     Fm2
.......................................................................uggacggaggauAgauaag...................   1     1     Fm2
.......................................................................uggacggaggauugauaaA...................   71    1     Fm2
.......................................................................uggacggaggUuugauaag...................   2     1     Fm2
.......................................................................uggacggaggauugauUag...................   1     1     Fm2
.......................................................................uggacggaggauugauaag...................   660   0     Fm2
.......................................................................uggacggUggauugauaag...................   1     1     Fm2
.......................................................................uggacggaggaCugauaagg..................   23    1     Fm2
.......................................................................uggacggagAauugauaagg..................   4     1     Fm2
.......................................................................uggacggaggauugauaagA..................   26    1     Fm2
.......................................................................uggacggaggauugauaagU..................   3     1     Fm2
.......................................................................uggacggaggauugauaagg..................   270   0     Fm2
.......................................................................Nggacggaggauugauaaggg.................   1     1     Fm2
.......................................................................uggacggagAauugauaaggg.................   15    1     Fm2
.......................................................................uggacggaggauugauaaggU.................   4     1     Fm2
.......................................................................uggacggaggauugauaUggg.................   2     1     Fm2
.......................................................................uggacggaggauugauaaggA.................   109   1     Fm2
.......................................................................uggacggaggaCugauaaggg.................   27    1     Fm2
.......................................................................uggacggaggauugauaaggg.................   352   0     Fm2
.......................................................................uggacggaggauugauaCggg.................   2     1     Fm2
.......................................................................uggacggaggauGgauaaggg.................   1     1     Fm2
.......................................................................uggacggaggCuugauaaggg.................   2     1     Fm2
.......................................................................uggacggagUauugauaaggg.................   1     1     Fm2
.......................................................................uggacggaUgauugauaaggg.................   1     1     Fm2
.......................................................................uggacggaggGuugauaaggg.................   1     1     Fm2
.......................................................................uggacggaggauugauGagggc................   34    1     Fm2
.......................................................................uggacggaggauGgauaagggc................   1     1     Fm2
.......................................................................Aggacggaggauugauaagggc................   5     1     Fm2
.......................................................................uUgacggaggauugauaagggc................   6     1     Fm2
.......................................................................uggacgCaggauugauaagggc................   4     1     Fm2
.......................................................................uggacgUaggauugauaagggc................   11    1     Fm2
.......................................................................uggacggaggauugCuaagggc................   1     1     Fm2
.......................................................................uggacggaCgauugauaagggc................   1     1     Fm2
.......................................................................uggacUgaggauugauaagggc................   15    1     Fm2
.......................................................................uggacggaggauugauaagUgc................   13    1     Fm2
.......................................................................uggacggUggauugauaagggc................   10    1     Fm2
.......................................................................uggacggaggauuUauaagggc................   7     1     Fm2
.......................................................................uggacgNaggauugauaagggc................   4     1     Fm2
.......................................................................uggacggaggauugauCagggc................   72    1     Fm2
.......................................................................uggacggaggUuugauaagggc................   48    1     Fm2
.......................................................................uggacggaggauugauaCgggc................   132   1     Fm2
.......................................................................uggacggaggauugauaaggAc................   8     1     Fm2
.......................................................................uggacggagCauugauaagggc................   1     1     Fm2
```

Star **Mature**

```
guguagugaagacugaagguaguucuuagcgaguccuucucuauccuucgccuaguuuauauuaaaauacuggacggaggauugauaagggcuugcggggacacgguc
......................................................................uggacggaUgauugauaagggc................ 14     1  Fm2
......................................................................uggGcggaggauugauaagggc................ 3      1  Fm2
......................................................................uggacggaggauugauaagAgc................ 7      1  Fm2
......................................................................ugAacggaggauugauaagggc................ 9      1  Fm2
......................................................................uggacggaggauugauaaAggc................ 3      1  Fm2
......................................................................uggacggaggauugauaagggc................ 33171  0  Fm2
......................................................................uCgacggaggauugauaagggc................ 3      1  Fm2
......................................................................uggacggaggauCgauaagggc................ 7      1  Fm2
......................................................................uggacggaggauugauaaggUc................ 10     1  Fm2
......................................................................uggacggaggauugauUagggc................ 44     1  Fm2
......................................................................uggacggaggauugGuaagggc................ 3      1  Fm2
......................................................................ugUacggaggauugauaagggc................ 6      1  Fm2
......................................................................uggacggaggauuCauaagggc................ 3      1  Fm2
......................................................................uggacAgaggauugauaagggc................ 14     1  Fm2
......................................................................uggaUggaggauugauaagggc................ 7      1  Fm2
......................................................................uggacggaggauuAauaagggc................ 9      1  Fm2
......................................................................uggacggaggauugauaagggG................ 6      1  Fm2
......................................................................uggacggaggauugauaUgggc................ 105    1  Fm2
......................................................................uggacggaggauugauaagggA................ 166    1  Fm2
......................................................................uggacNgaggauugauaagggc................ 4      1  Fm2
......................................................................uggacggaggauugauaaggCc................ 1      1  Fm2
......................................................................uggacggGggauugauaagggc................ 128    1  Fm2
......................................................................uggacggaggaGugauaagggc................ 3      1  Fm2
......................................................................Cggacggaggauugauaagggc................ 3      1  Fm2
......................................................................ugCacggaggauugauaagggc................ 2      1  Fm2
......................................................................uggacggaggaCugauaagggc................ 1032   1  Fm2
......................................................................uggacggaggauugUuaagggc................ 3      1  Fm2
......................................................................uggacggaggauugaCaagggc................ 2      1  Fm2
......................................................................uggacggaggauugaGaagggc................ 1      1  Fm2
......................................................................uggaGggaggauugauaagggc................ 2      1  Fm2
......................................................................uggacggaggauugauaGgggc................ 114    1  Fm2
......................................................................uggacggaggauugauaagggU................ 38     1  Fm2
......................................................................uggUcggaggauugauaagggc................ 1      1  Fm2
......................................................................ugNacggaggauugauaagggc................ 1      1  Fm2
......................................................................uggCcggaggauugauaagggc................ 4      1  Fm2
......................................................................uggacggaggauugaAaagggc................ 1      1  Fm2
......................................................................uggacCgaggauugauaagggc................ 2      1  Fm2
......................................................................uggacggaggaAugauaagggc................ 4      1  Fm2
......................................................................uggacggagUauugauaagggc................ 7      1  Fm2
......................................................................uAgacggaggauugauaagggc................ 22     1  Fm2
......................................................................Nggacggaggauugauaagggc................ 18     1  Fm2
......................................................................uggacggaggGuugauaagggc................ 33     1  Fm2
......................................................................uggacggagAauugauaagggc................ 70     1  Fm2
......................................................................uggacgAaggauugauaagggc................ 2      1  Fm2
......................................................................uggacggaggCuugauaagggc................ 36     1  Fm2
......................................................................uggacggaggauugauaagCgc................ 2      1  Fm2
......................................................................uggacggaAgauugauaagggc................ 1      1  Fm2
......................................................................uggacggaggauugauaaUggc................ 4      1  Fm2
......................................................................uggaAggaggauugauaagggc................ 1      1  Fm2
......................................................................uggacggCggauugauaagggc................ 3      1  Fm2
......................................................................Gggacggaggauugauaagggc................ 1      1  Fm2
......................................................................uggacggaggaCugauaagggcu............... 37     1  Fm2
......................................................................uggacggaggauugauaagggcu............... 987    0  Fm2
......................................................................uggacggagAauugauaagggcu............... 2      1  Fm2
......................................................................uggacggaggauugauaagggcG............... 39     1  Fm2
......................................................................uggacggaggauugauaagggcC............... 5      1  Fm2
......................................................................uggacUgaggauugauaagggcu............... 1      1  Fm2
......................................................................uggacggaggGuugauaagggcu............... 1      1  Fm2
......................................................................uggaAggaggauugauaagggcu............... 1      1  Fm2
......................................................................Nggacggaggauugauaagggcu............... 1      1  Fm2
......................................................................uggacggaggauugauaUgggcu............... 2      1  Fm2
......................................................................uUgacggaggauugauaagggcu............... 1      1  Fm2
......................................................................uggacggaggauugauaagggAu............... 10     1  Fm2
......................................................................uggacggaggauuAauaagggcu............... 1      1  Fm2
......................................................................uggacggCggauugauaagggcu............... 1      1  Fm2
......................................................................uggacggaggauugauCagggcu............... 2      1  Fm2
......................................................................uggacggaggauugaGaagggcu............... 1      1  Fm2
......................................................................uggacgUaggauugauaagggcu............... 1      1  Fm2
......................................................................uggacggaggCuugauaagggcu............... 1      1  Fm2
......................................................................uggacggaggauugauaagggcA............... 295    1  Fm2
```

```
                                   Star                               Mature
guguagugaagacugaagguaguucuuagcgaguccuucucuauccuucgccuaguuuauauuaaaauacuggacggaggauugauaagggcuugcggggacacgguc
......................................................................uggacggaggauugauaCgggcu...............    4    1    Fm2
......................................................................uggacAgaggauugauaagggcu...............    1    1    Fm2
......................................................................uggacggaggauugauUagggcu...............    1    1    Fm2
......................................................................uggacggaggauugauGagggcu...............    1    1    Fm2
......................................................................uggacggaggauugauaGgggcu...............    1    1    Fm2
......................................................................uggacggaggaCugauaagggcuu..............    2    1    Fm2
......................................................................uggacggaggauugauaagggcAu..............    33   1    Fm2
......................................................................uggacggaggauugauaagggUuu..............    20   1    Fm2
......................................................................uggacggaggauugauUagggcuu..............    1    1    Fm2
......................................................................uggacggaggauugauaUgggcuu..............    1    1    Fm2
......................................................................uggacggaggauugauaagggcuu..............    73   0    Fm2
......................................................................uggacggaggGuugauaagggcuu..............    1    1    Fm2
......................................................................uggacggaggauugauaagggcCu..............    8    1    Fm2
......................................................................uggacggaggauugauaagggcuuU.............    94   1    Fm2
.......................................................................ggacggaggaCugauaagg..................    1    1    Fm2
.......................................................................ggacggagAauugauaagggc................    2    1    Fm2
.......................................................................ggacggaggauugauaagggc................    5    0    Fm2
.......................................................................ggacggaggauugauaagggcA...............    2    1    Fm2
..........................................................................cggaggauugauaagggc................    3    0    Fm2

..................................ccuucucuauccuuUgccu.......................................................    1    1    Mm2
..................................ccuucucuauccuucgccu.......................................................    6    0    Mm2
..................................ccuucucuauccuucgccuagu....................................................    39   0    Mm2
..................................ccuucucuauccuucgcGuagu....................................................    1    1    Mm2
..................................ccuucucuauccuucgccuaguu...................................................    1    0    Mm2
..................................ccuucucuauccuucgccuaguA...................................................    1    1    Mm2
...................................cuucucuauccuucgccua......................................................    1    0    Mm2
...................................cuucucuauUcuucgccuagu....................................................    1    1    Mm2
...................................cuucucuauccuucgccuagu....................................................    119  0    Mm2
...................................cAucucuauccuucgccuagu....................................................    1    1    Mm2
...................................cuucucuauccuucgcAuagu....................................................    1    1    Mm2
...................................cuucucuauccuucgccGagu....................................................    1    1    Mm2
...................................cuucucuaucGuucgccuagu....................................................    1    1    Mm2
...................................cuucucuauccuucgccuCgu....................................................    1    1    Mm2
...................................cuucucuauccuucgccCagu....................................................    1    1    Mm2
...................................cuucucuauccuucgccuaguu...................................................    5    0    Mm2
...................................cuucucuauccuucgccuaguA...................................................    3    1    Mm2
...................................cuucucuauccuuUgccuaguu...................................................    1    1    Mm2
...................................cuucucuauccuucgccuaguuu..................................................    2    0    Mm2
....................................uucucuauccuucgccuaguuu..................................................    3    0    Mm2
....................................uucucuauccuucgccuaguuua.................................................    3    0    Mm2
...............................................................aaaauacuggacggaggauugauaagggc................    1    0    Mm2
...................................................................uacuggacggaggauugau......................    2    0    Mm2
...................................................................uacuggacggaggauugauU.....................    1    1    Mm2
...................................................................uacuggacggaggauugauaa....................    1    0    Mm2
...................................................................uacuggacggaggauugauaag...................    5    0    Mm2
....................................................................acuggacggaggauugauaag...................    10   0    Mm2
....................................................................acuggacggaggauugauaagg..................    2    0    Mm2
....................................................................acuggacggaggauugauaaggU.................    1    1    Mm2
....................................................................Ucuggacggaggauugauaagggc................    2    1    Mm2
.....................................................................cuggacggaggauCgauaagggc................    1    1    Mm2
.....................................................................cuggacggaggauugauaagggc................    11   0    Mm2
.....................................................................cuggacggagUauugauaagggc................    1    1    Mm2
.....................................................................cuggacggaggauugauaagggcu...............    1    0    Mm2
.....................................................................cuggacggaggaCugauaagggcuu..............    1    1    Mm2
......................................................................uggacggaggauugauaa....................    10   0    Mm2
......................................................................uggacggaggauugaAaa....................    1    1    Mm2
......................................................................uggacggaggUuugauaag...................    1    1    Mm2
......................................................................uggacggaggaCugauaag...................    12   1    Mm2
......................................................................uggacggaggauugauaag...................    322  0    Mm2
......................................................................uggacggaggauugauaaA...................    19   1    Mm2
......................................................................uggacggaggGuugauaag...................    1    1    Mm2
......................................................................uggacggaggauugauaUg...................    4    1    Mm2
......................................................................uggacggaggauugauaaU...................    4    1    Mm2
......................................................................uggacggagAauugauaagg..................    3    1    Mm2
......................................................................uggacggaggGuugauaagg..................    1    1    Mm2
......................................................................uggacggaggauugauaagU..................    2    1    Mm2
......................................................................uggacggaggauugauaagg..................    105  0    Mm2
......................................................................uggacggaggauugauaagA..................    13   1    Mm2
......................................................................uggCcggaggauugauaagg..................    1    1    Mm2
```

Star Mature

```
guguagugaagacugaagguaguucuuagcgagucсuucucuauccuucgccuaguuuauauuaaaauacuggacggaggauugauaagggcuugcggggacacgguc
......................................................................uggacggaggaCugauaagg..................  24     1  Mm2
......................................................................uggacggGggauugauaaggg.................  14     1  Mm2
......................................................................uggacggaggauugauaCggg.................  1      1  Mm2
......................................................................uggacggagAauugauaaggg.................  16     1  Mm2
......................................................................uggacggaggauugauaaggC.................  4      1  Mm2
......................................................................uggCcggaggauugauaaggg.................  2      1  Mm2
......................................................................uggacggaggauugauaUggg.................  3      1  Mm2
......................................................................uggacggaggauugauaGggg.................  1      1  Mm2
......................................................................uggacUgaggauugauaaggg.................  1      1  Mm2
......................................................................uggacggaggauugauaaggg.................  518    0  Mm2
......................................................................uggacggaggauuAauaaggg.................  1      1  Mm2
......................................................................uggacggaggUuugauaaggg.................  2      1  Mm2
......................................................................uggacggaggauugauaaggA.................  52     1  Mm2
......................................................................uggacggaggauugauaaUgg.................  1      1  Mm2
......................................................................uggacggaggauugauaaggU.................  2      1  Mm2
......................................................................uggacggaggaCugauaaggg.................  50     1  Mm2
......................................................................uUgacggaggauugauaagggc................  3      1  Mm2
......................................................................uggacgUaggauugauaagggc................  5      1  Mm2
......................................................................uggacggaggauugGuaagggc................  2      1  Mm2
......................................................................uggacggaggauugauaaggUc................  4      1  Mm2
......................................................................uggacggaggauCgauaagggc................  4      1  Mm2
......................................................................uggaAggaggauugauaagggc................  1      1  Mm2
......................................................................uggacAgaggauugauaagggc................  4      1  Mm2
......................................................................ugAacggaggauugauaagggc................  5      1  Mm2
......................................................................uggacggaggauugauaagggA................  158    1  Mm2
......................................................................uggacggaggauugaCaagggc................  2      1  Mm2
......................................................................Cggacggaggauugauaagggc................  1      1  Mm2
......................................................................uggacggaggauugauUagggc................  21     1  Mm2
......................................................................uggacggaggauugauaagAgc................  3      1  Mm2
......................................................................uggacggGggauugauaagggc................  138    1  Mm2
......................................................................uggacggaggauugauaagggG................  3      1  Mm2
......................................................................uggacggaggUuugauaagggc................  48     1  Mm2
......................................................................uggacggaggauugauaGgggc................  55     1  Mm2
......................................................................uggacUgaggauugauaagggc................  4      1  Mm2
......................................................................ugUacggaggauugauaagggc................  1      1  Mm2
......................................................................Aggacggaggauugauaagggc................  4      1  Mm2
......................................................................uggacggaggauugauaUgggc................  80     1  Mm2
......................................................................uggacggaggCuugauaagggc................  30     1  Mm2
......................................................................uggacggaggauugUuaagggc................  3      1  Mm2
......................................................................uggacggaggauugauaCgggc................  86     1  Mm2
......................................................................Nggacggaggauugauaagggc................  8      1  Mm2
......................................................................uggacggaggGuugauaagggc................  36     1  Mm2
......................................................................uggacggUggauugauaagggc................  11     1  Mm2
......................................................................uggacgNaggauugauaagggc................  1      1  Mm2
......................................................................uggGcggaggauugauaagggc................  6      1  Mm2
......................................................................ugCacggaggauugauaagggc................  1      1  Mm2
......................................................................uggacggaggauugauaaUggc................  3      1  Mm2
......................................................................uggacggaUgauugauaagggc................  2      1  Mm2
......................................................................uggacggCggauugauaagggc................  3      1  Mm2
......................................................................uggacggaggauugauaaggCc................  4      1  Mm2
......................................................................uggacggaCgauugauaagggc................  1      1  Mm2
......................................................................uCgacggaggauugauaagggc................  1      1  Mm2
......................................................................uggacggaggaAugauaagggc................  4      1  Mm2
......................................................................uggacggaggauuCauaagggc................  3      1  Mm2
......................................................................uggacggagCauugauaagggc................  1      1  Mm2
......................................................................uggacggaggauugauaagggU................  43     1  Mm2
......................................................................uggacggaggauuUauaagggc................  3      1  Mm2
......................................................................uggacggaggaCugauaagggc................  998    1  Mm2
......................................................................uggCcggaggauugauaagggc................  6      1  Mm2
......................................................................uggacggagUauugauaagggc................  3      1  Mm2
......................................................................uggaGggaggauugauaagggc................  4      1  Mm2
......................................................................uggacggaggauugauaagUgc................  4      1  Mm2
......................................................................uggacggaggauugauCagggc................  47     1  Mm2
......................................................................uggacgAaggauugauaagggc................  1      1  Mm2
......................................................................uggacCgaggauugauaagggc................  1      1  Mm2
......................................................................uggacggaggauugauaaAggc................  1      1  Mm2
......................................................................uggacggagAauugauaagggc................  79     1  Mm2
......................................................................uggacggaggauugauaagggc................  20814  0  Mm2
......................................................................uAgacggaggauugauaagggc................  8      1  Mm2
......................................................................uggaUggaggauugauaagggc................  4      1  Mm2
```

```
                                   Star                           Mature
guguagugaagacugaagguaguucuuagcgaguccuucucuauccuucgccuaguuuauauuaaaauacuggacggaggauugauaagggcuugcggggacacgguc
......................................................................uggacNgaggauugauaagggc................  3     1   Mm2
......................................................................uggUcggaggauugauaagggc................  4     1   Mm2
......................................................................Gggacggaggauugauaagggc................  1     1   Mm2
......................................................................uggacggaggauGgauaagggc................  1     1   Mm2
......................................................................uggacggaggauAgauaagggc................  1     1   Mm2
......................................................................uggacggaAgauugauaagggc................  4     1   Mm2
......................................................................uggacggaggauuAauaagggc................  7     1   Mm2
......................................................................uggacggaggauugauGagggc................  30    1   Mm2
......................................................................uggacggaggauugauaagCgc................  1     1   Mm2
......................................................................uggacggaggauugauaaggAc................  5     1   Mm2
......................................................................uggacgCaggauugauaagggc................  1     1   Mm2
......................................................................uggacggaggauugauUagggcu...............  4     1   Mm2
......................................................................uggacggaggauugauCagggcu...............  5     1   Mm2
......................................................................uggacggaggaAugauaagggcu...............  1     1   Mm2
......................................................................uggacggaggauugauaagggcA...............  301   1   Mm2
......................................................................uggacggaggaCugauaagggcu...............  45    1   Mm2
......................................................................uggacggGggauugauaagggcu...............  2     1   Mm2
......................................................................uggacggaggauuCauaagggcu...............  1     1   Mm2
......................................................................uggacggaggauugauGagggcu...............  2     1   Mm2
......................................................................uggacggaggauugauaagggcG...............  26    1   Mm2
......................................................................ugUacggaggauugauaagggcu...............  1     1   Mm2
......................................................................uggacggaggUuugauaagggcu...............  3     1   Mm2
......................................................................uggacggaggauugauaagggcC...............  3     1   Mm2
......................................................................uggacggaggauugauaagggAu...............  4     1   Mm2
......................................................................uggacggagUauugauaagggcu...............  1     1   Mm2
......................................................................uggacggaggauugauaUgggcu...............  1     1   Mm2
......................................................................uggacggaggauugauaaAggcu...............  1     1   Mm2
......................................................................uggacggaggauugauaCgggcu...............  6     1   Mm2
......................................................................uggacggagAauugauaagggcu...............  3     1   Mm2
......................................................................uggaAggaggauugauaagggcu...............  1     1   Mm2
......................................................................uggacggaggauugauaagggcu...............  1031  0   Mm2
......................................................................uggacggaggauugauaagggUu...............  1     1   Mm2
......................................................................uggacggaggauugauaGgggcu...............  2     1   Mm2
......................................................................uggacggUggauugauaagggcu...............  1     1   Mm2
......................................................................uggacggaggGuugauaagggcu...............  3     1   Mm2
......................................................................uggacggaggauugauaagggcuA..............  4     1   Mm2
......................................................................uggacggaggauugauaCgggcuu..............  1     1   Mm2
......................................................................uggacggaggauugauaagggcAu..............  47    1   Mm2
......................................................................uggacggGggauugauaagggcuu..............  1     1   Mm2
......................................................................uggacggaggauugauaUgggcuu..............  1     1   Mm2
......................................................................uggacggaggauugauaagggUuu..............  2     1   Mm2
......................................................................uggacggaggauugauCagggcuu..............  1     1   Mm2
......................................................................uggacggUggauugauaagggcuu..............  1     1   Mm2
......................................................................Nggacggaggauugauaagggcuu..............  2     1   Mm2
......................................................................uggCcggaggauugauaagggcuu..............  2     1   Mm2
......................................................................uggacggaggauugauaagggcuC..............  1     1   Mm2
......................................................................uggacggaggaCugauaagggcuu..............  7     1   Mm2
......................................................................uggacNgaggauugauaagggcuu..............  1     1   Mm2
......................................................................uggacggagAauugauaagggcuu..............  1     1   Mm2
......................................................................uggacggaggauugauUagggcuu..............  1     1   Mm2
......................................................................uggacggaggauugauaagggcuG..............  1     1   Mm2
......................................................................uggacggaggauugauaagggcCu..............  1     1   Mm2
......................................................................uggacggaggauugauaagggcuu..............  177   0   Mm2
......................................................................uggacggaggauugauaagggcuuU.............  105   1   Mm2
......................................................................uggacggaggauugauaagggcuuA.............  1     1   Mm2
.......................................................................Ugacggaggauugauaagggc................  1     1   Mm2
.......................................................................ggacggaggauugauaagggc................  7     0   Mm2
.......................................................................ggacggagAauugauaagggc................  3     1   Mm2
.......................................................................ggacggaggauugauaCgggc................  1     1   Mm2
.......................................................................ggacggaggaCugauaagggcu...............  2     1   Mm2
..........................................................................Uggaggauugauaagggc................  1     1   Mm2

..................................ccuucucuauccuucgccu.......................................................  12    0   Mm1
..................................ccuucucuauccuucgcUuagu....................................................  2     1   Mm1
..................................Ncuucucuauccuucgccuagu....................................................  1     1   Mm1
..................................ccuucucuauccuucgccuagu....................................................  37    0   Mm1
...................................cuucucuauccuucgccuagu....................................................  4     0   Mm1
.....................................................................cuggacggaggauugauaagggc................  8     0   Mm1
......................................................................uggacggaggauugauaa....................  6     0   Mm1
......................................................................uggacggaggauuAauaa....................  1     1   Mm1
```

Star Mature

```
guguagugaagacugaagguaguucuuagcgaguccuucucuauccuucgccuaguuuauauuaaaauacuggacggaggauugauaagggcuugcggggacacgguc
......................................................................uggacggaggauugaCaa....................	1	1	Mm1
......................................................................uggacggaggauuAauaag...................	1	1	Mm1
......................................................................uggacggaggauugauaag...................	216	0	Mm1
......................................................................uggacggaggaCugauaag...................	6	1	Mm1
......................................................................Cggacggaggauugauaag...................	1	1	Mm1
......................................................................uggacggaggauugauaCg...................	1	1	Mm1
......................................................................uggacggaggauugauaaA...................	2	1	Mm1
......................................................................uggacggaggauugauGag...................	1	1	Mm1
......................................................................uggacggaggauugauCag...................	3	1	Mm1
......................................................................uggacggaggUuugauaag...................	1	1	Mm1
......................................................................uggacggagAauugauaagg..................	14	1	Mm1
......................................................................uggacggaggauugauaagg..................	83	0	Mm1
......................................................................uggacggaggauugauaagU..................	3	1	Mm1
......................................................................uggacggaggauugauCagg..................	1	1	Mm1
......................................................................uggacggaggauugauaagA..................	7	1	Mm1
......................................................................uggacggaggauugauaUgg..................	2	1	Mm1
......................................................................uggacggaggaCugauaagg..................	8	1	Mm1
......................................................................uggacggaggUuugauaagg..................	1	1	Mm1
......................................................................uggacggaggauugauUagg..................	1	1	Mm1
......................................................................uggacggaggaAugauaaggg.................	1	1	Mm1
......................................................................uggacggaggauugauaGggg.................	1	1	Mm1
......................................................................ugAacggaggauugauaaggg.................	1	1	Mm1
......................................................................uggacggagAauugauaaggg.................	1	1	Mm1
......................................................................uggacggaggaCugauaaggg.................	3	1	Mm1
......................................................................uggacggaggauugauaaggg.................	192	0	Mm1
......................................................................uggacggGggauugauaaggg.................	11	1	Mm1
......................................................................uggacggaggauugauaUggg.................	1	1	Mm1
......................................................................uggacggaggauugauaaggA.................	23	1	Mm1
......................................................................uggacggaggauugauaCggg.................	2	1	Mm1
......................................................................uggacggaggauugauaaggC.................	1	1	Mm1
......................................................................uggacggaggGuugauaaggg.................	1	1	Mm1
......................................................................uggacggaggauugauGagggc................	42	1	Mm1
......................................................................uggacggaggauugGuaagggc................	2	1	Mm1
......................................................................uggaGggaggauugauaagggc................	2	1	Mm1
......................................................................uggacggaggauugauaUgggc................	71	1	Mm1
......................................................................ugUacggaggauugauaagggc................	3	1	Mm1
......................................................................Cggacggaggauugauaagggc................	4	1	Mm1
......................................................................uggacggaggauugauaaUggc................	1	1	Mm1
......................................................................uggacggaggauCgauaagggc................	2	1	Mm1
......................................................................uggacggaggauugauaaCggc................	1	1	Mm1
......................................................................uggacggaggauugauaagAgc................	7	1	Mm1
......................................................................uggacggCggauugauaagggc................	1	1	Mm1
......................................................................uUgacggaggauugauaagggc................	4	1	Mm1
......................................................................uggUcggaggauugauaagggc................	4	1	Mm1
......................................................................uggacggaggauugauaagUgc................	9	1	Mm1
......................................................................uggacggaggauugauaGgggc................	86	1	Mm1
......................................................................uggacggaggUuugauaagggc................	34	1	Mm1
......................................................................uggacggaggauugauaaggAc................	1	1	Mm1
......................................................................uggacAgaggauugauaagggc................	3	1	Mm1
......................................................................uggacggaggaCugauaagggc................	251	1	Mm1
......................................................................uggacgUaggauugauaagggc................	4	1	Mm1
......................................................................uggacggaggauugUuaagggc................	5	1	Mm1
......................................................................uggacggaggauugauaagggG................	6	1	Mm1
......................................................................uggacgAaggauugauaagggc................	1	1	Mm1
......................................................................ugAacggaggauugauaagggc................	4	1	Mm1
......................................................................uAgacggaggauugauaagggc................	10	1	Mm1
......................................................................uggacggaggauugauaagCgc................	3	1	Mm1
......................................................................uggacggaggauugauaagggU................	24	1	Mm1
......................................................................uggacggaggGuugauaagggc................	25	1	Mm1
......................................................................uggacggaggauuAauaagggc................	3	1	Mm1
......................................................................uggacUgaggauugauaagggc................	3	1	Mm1
......................................................................uggacggaggCuugauaagggc................	18	1	Mm1
......................................................................uggCcggaggauugauaagggc................	3	1	Mm1
......................................................................uggacggaggauugauaCgggc................	80	1	Mm1
......................................................................uggacggaCgauugauaagggc................	1	1	Mm1
......................................................................uggacggaggauugauaagggc................	21879	0	Mm1
......................................................................uggacggaggauAgauaagggc................	4	1	Mm1
......................................................................uggacggaUgauugauaagggc................	3	1	Mm1
......................................................................uggacggaggauGgauaagggc................	1	1	Mm1
......................................................................uggacggaggauugauaaggUc................	7	1	Mm1
```

```
                                                   Star                                  Mature
guguagugaagacugaagguaguucuuagcgagucCUUCUCUAUCCUUCGCCUAGUuuauauuaaaauacUGGACGGAGGAUUGAUAAGGGCuugcggggacacgguc
```

```
                                                   Star                                  Mature
guguagugaagacugaagguaguucuuagcgaguccuucucuauccuucgccuaguuuauauuaaaauacuggacggaggauugauaagggcuugcggggacacgguc
......................................................................uggGcggaggauugauaagggc................    6     1   Mm1
......................................................................uggacggagCauugauaagggc................    2     1   Mm1
......................................................................uggacggaggauugauUagggc................    31    1   Mm1
......................................................................uggacggaggauuCauaagggc................    1     1   Mm1
......................................................................uggacgNaggauugauaagggc................    2     1   Mm1
......................................................................uggacggaggaAugauaagggc................    7     1   Mm1
......................................................................Nggacggaggauugauaagggc................    14    1   Mm1
......................................................................uggacggaggauugauaagggA................    216   1   Mm1
......................................................................uggacggagAauugauaagggc................    20    1   Mm1
......................................................................uggacggaggauugauaaAggc................    4     1   Mm1
......................................................................Aggacggaggauugauaagggc................    1     1   Mm1
......................................................................uggacggGggauugauaagggc................    72    1   Mm1
......................................................................uggaUggaggauugauaagggc................    4     1   Mm1
......................................................................uggacggaggauuUauaagggc................    1     1   Mm1
......................................................................uggacggUggauugauaagggc................    8     1   Mm1
......................................................................uggaAggaggauugauaagggc................    2     1   Mm1
......................................................................uggacggaggauugauCagggc................    53    1   Mm1
......................................................................uggacggaggauugauaagggcC...............    5     1   Mm1
......................................................................uggacggaggauugauaagggcG...............    5     1   Mm1
......................................................................uggacggaggauugauGagggcu...............    1     1   Mm1
......................................................................uggacggaggauugGuaagggcu...............    1     1   Mm1
......................................................................uggacggaggauugauaagggUu...............    10    1   Mm1
......................................................................uggacggaggaAugauaagggcu...............    1     1   Mm1
......................................................................uggacggaggUuugauaagggcu...............    4     1   Mm1
......................................................................uggacggaggauugauCagggcu...............    3     1   Mm1
......................................................................uggacggagUauugauaagggcu...............    1     1   Mm1
......................................................................uggacgNaggauugauaagggcu...............    1     1   Mm1
......................................................................uggacggUggauugauaagggcu...............    2     1   Mm1
......................................................................uggacggaggauugauaGgggcu...............    3     1   Mm1
......................................................................uggacggaggauugauaCgggcu...............    4     1   Mm1
......................................................................uggacggaggauugauaagggcu...............    1340  0   Mm1
......................................................................uggacAgaggauugauaagggcu...............    1     1   Mm1
......................................................................uggacggaggauugauUagggcu...............    4     1   Mm1
......................................................................uggacggaggauugauaUgggcu...............    3     1   Mm1
......................................................................uggacggaggauugauaagggcA...............    424   1   Mm1
......................................................................uggacggaggauugauaaggUcu...............    1     1   Mm1
......................................................................uggacggaggCuugauaagggcu...............    1     1   Mm1
......................................................................uggacggaggauuAauaagggcu...............    1     1   Mm1
......................................................................uggacggaggauugaGaagggcu...............    1     1   Mm1
......................................................................uggacggaggaCugauaagggcu...............    14    1   Mm1
......................................................................uggacggaggauugauaaAggcu...............    1     1   Mm1
......................................................................uggacggaggauugauaagggcAu..............    71    1   Mm1
......................................................................uggacggaggauugauaagggcuu..............    118   0   Mm1
......................................................................uggacggaggauugauaagggcuG..............    1     1   Mm1
......................................................................uggacggaggauugauaagggcGu..............    1     1   Mm1
......................................................................uggacggagAauugauaagggcuu..............    1     1   Mm1
......................................................................uggacggaggauugauaagggcuA..............    23    1   Mm1
......................................................................uggacggaggauugauaCgggcuu..............    1     1   Mm1
......................................................................uggacggaggauugauaagggUuu..............    4     1   Mm1
......................................................................uggacggaggauugauaagggcAug.............    1     1   Mm1
......................................................................uggacggaggauugauaagggcuuU.............    34    1   Mm1
.......................................................................Ugacggaggauugauaagggc................    1     1   Mm1
.......................................................................ggacggaggauugauaagggc................    4     0   Mm1
.......................................................................ggacggaggaCugauaagggc................    2     1   Mm1

..................................ccuucucuauccuucgccu.......................................................    16    0   Fm3
..................................ccuucucuauccuucgccuag.....................................................    8     0   Fm3
..................................ccuucucuauccuucgAcuagu....................................................    1     1   Fm3
..................................ccuucucuauccuucgcUuagu....................................................    1     1   Fm3
..................................ccuucucuauccuucgUcuagu....................................................    1     1   Fm3
..................................ccuucucuaCccuucgccuagu....................................................    1     1   Fm3
..................................ccuucucuauccuucgccuagu....................................................    94    0   Fm3
...................................cuucucuauccuucgccGagu....................................................    1     1   Fm3
...................................cuucucuauccuucgccuagu....................................................    27    0   Fm3
...................................cuucucuauccuucgccuaguu...................................................    3     0   Fm3
...................................cuucucuauccuucgccCaguuu..................................................    1     1   Fm3
...................................................................uacuggacggaggauugau......................    4     0   Fm3
...................................................................uacuggacggaggauugaua.....................    3     0   Fm3
...................................................................uacuggacggGggauugauaa....................    1     1   Fm3
...................................................................uacuggacggaUgauugauaa....................    1     1   Fm3
```

```
                                   Star                              Mature
guguagugaagacugaagguaguucuuagcgaguccuucucuauccuucgccuaguuuauauuaaaauacuggacggaggauugauaagggcuugcggggacacgguc
...................................................................uacuggacggaggauugauaa....................  26     0  Fm3
...................................................................uacuggacggaggauugauaag...................  59     0  Fm3
...................................................................uacuggacggaggauugauaUg...................  1      1  Fm3
...................................................................uacuggacggaggauugauaaU...................  2      1  Fm3
...................................................................uacuggacggaggauuCauaag...................  1      1  Fm3
...................................................................uacuggacggaggauugauaagU..................  1      1  Fm3
....................................................................acuggacggaggauugauaa....................  1      0  Fm3
....................................................................acuggacggaggauugauaag...................  21     0  Fm3
....................................................................acGggacggaggauugauaag...................  1      1  Fm3
....................................................................acuggacggaggauugaGaag...................  1      1  Fm3
....................................................................acuggacggaCgauugauaagg..................  1      1  Fm3
....................................................................acuggacggaggauugauaagg..................  34     0  Fm3
....................................................................Ucuggacggaggauugauaagggc................  2      1  Fm3
.....................................................................cuggacggaggCuugauaagg..................  1      1  Fm3
.....................................................................cuggacggaggaCugauaagg..................  1      1  Fm3
.....................................................................cuggacggaggauugauaagg..................  4      0  Fm3
.....................................................................cuggacggaggaCugauaaggg.................  2      1  Fm3
.....................................................................cuggacggaggauugauaaggg.................  2      0  Fm3
.....................................................................cuggacggaggauugauaagggA................  1      1  Fm3
.....................................................................cuggacggaggauugauaagggc................  37     0  Fm3
.....................................................................cuggacggaggauugauaagggcA...............  4      1  Fm3
......................................................................Aggacggaggauugauaa....................  1      1  Fm3
......................................................................uggacggaggauugaAaa....................  38     1  Fm3
......................................................................uggacggUggauugauaa....................  2      1  Fm3
......................................................................uggacggaggauugauaa....................  742    0  Fm3
......................................................................uggacggaggauugauaU....................  15     1  Fm3
......................................................................uggacggaggauugauUa....................  9      1  Fm3
......................................................................uggacAgaggauugauaa....................  1      1  Fm3
......................................................................uAgacggaggauugauaa....................  1      1  Fm3
......................................................................uggacggaggGuugauaa....................  2      1  Fm3
......................................................................uggacggaggauugauGa....................  1      1  Fm3
......................................................................uggacgUaggauugauaa....................  1      1  Fm3
......................................................................uggacggaggaCugauaa....................  15     1  Fm3
......................................................................uggacggaAgauugauaa....................  1      1  Fm3
......................................................................uggacggagAauugauaa....................  1      1  Fm3
......................................................................uggacggaggauugauaC....................  4      1  Fm3
......................................................................uggacggaggauugauCa....................  2      1  Fm3
......................................................................uggaAggaggauugauaag...................  2      1  Fm3
......................................................................uggacggaggauuUauaag...................  2      1  Fm3
......................................................................uggacggCggauugauaag...................  2      1  Fm3
......................................................................uggacggagUauugauaag...................  1      1  Fm3
......................................................................uggacggaggCuugauaag...................  16     1  Fm3
......................................................................uggacggaggaAugauaag...................  5      1  Fm3
......................................................................uggacgUaggauugauaag...................  11     1  Fm3
......................................................................uggacggaggauugauaUg...................  48     1  Fm3
......................................................................ugAacggaggauugauaag...................  1      1  Fm3
......................................................................uggacAgaggauugauaag...................  3      1  Fm3
......................................................................ugUacggaggauugauaag...................  5      1  Fm3
......................................................................uggacggaggaGugauaag...................  1      1  Fm3
......................................................................uggacUgaggauugauaag...................  4      1  Fm3
......................................................................uggaUggaggauugauaag...................  5      1  Fm3
......................................................................Nggacggaggauugauaag...................  3      1  Fm3
......................................................................uggGcggaggauugauaag...................  3      1  Fm3
......................................................................uggacggaggauuAauaag...................  5      1  Fm3
......................................................................uggacggaggauugauaaA...................  1072   1  Fm3
......................................................................uggacggaggauuCauaag...................  1      1  Fm3
......................................................................uggacggaggauugauGag...................  28     1  Fm3
......................................................................uAgacggaggauugauaag...................  6      1  Fm3
......................................................................uggacggagAauugauaag...................  23     1  Fm3
......................................................................uggacggGggauugauaag...................  4      1  Fm3
......................................................................uCgacggaggauugauaag...................  5      1  Fm3
......................................................................uggacggaggaCugauaag...................  72     1  Fm3
......................................................................uggacggaggauugauaag...................  14379  0  Fm3
......................................................................uggCcggaggauugauaag...................  6      1  Fm3
......................................................................uggacggaggauugauaCg...................  44     1  Fm3
......................................................................uggacggaggauugauCag...................  34     1  Fm3
......................................................................uggacggaggGuugauaag...................  17     1  Fm3
......................................................................uggacggaggauugaCaag...................  1      1  Fm3
......................................................................uggacggaggauugGuaag...................  4      1  Fm3
......................................................................uggacNgaggauugauaag...................  3      1  Fm3
```

Star Mature

guguagugaagacugaagguaguucuuagcgaguccuucucuauccuucgccuaguuuauauuaaaauacuggacggaggauugauaagggcuugcggggacacgguc			
....uggacggaggauugauUag...................	15	1	Fm3
....uggacggaggauugCuaag...................	2	1	Fm3
....uUgacggaggauugauaag...................	6	1	Fm3
....uggacggaggauugUuaag...................	3	1	Fm3
....uggacggaggUuugauaag...................	29	1	Fm3
....uggacggaggauugauaaU...................	150	1	Fm3
....uggacggUggauugauaag...................	7	1	Fm3
....uggacggaUgauugauaag...................	7	1	Fm3
....uggacggaggauAgauaag...................	1	1	Fm3
....ugCacggaggauugauaag...................	3	1	Fm3
....uggacggaggauugauaagA..................	556	1	Fm3
....uggacggaggauugauaaUg..................	2	1	Fm3
....uggaUggaggauugauaagg..................	1	1	Fm3
....uggacggaggaGugauaagg..................	1	1	Fm3
....uggacggaggauugauaUgg..................	11	1	Fm3
....uggacggaggUuugauaagg..................	19	1	Fm3
....uggacAgaggauugauaagg..................	1	1	Fm3
....uAgacggaggauugauaagg..................	2	1	Fm3
....uggacggaggauugGuaagg..................	2	1	Fm3
....uggacggGggauugauaagg..................	1	1	Fm3
....Nggacggaggauugauaagg..................	4	1	Fm3
....uggacggaggauugauaagU..................	108	1	Fm3
....Aggacggaggauugauaagg..................	1	1	Fm3
....uggacggaggauuAauaagg..................	1	1	Fm3
....uggacggagUauugauaagg..................	1	1	Fm3
....uggacggaggaAugauaagg..................	2	1	Fm3
....uggCcggaggauugauaagg..................	2	1	Fm3
....uggacggaggaCugauaagg..................	235	1	Fm3
....ugUacggaggauugauaagg..................	3	1	Fm3
....uggacUgaggauugauaagg..................	2	1	Fm3
....uggacggaggauugaAaagg..................	1	1	Fm3
....uggacggaggGuugauaagg..................	4	1	Fm3
....uggacggaggauugauaGgg..................	8	1	Fm3
....uUgacggaggauugauaagg..................	1	1	Fm3
....uggacggaggauugauaCgg..................	21	1	Fm3
....uggacgUaggauugauaagg..................	2	1	Fm3
....Cggacggaggauugauaagg..................	1	1	Fm3
....uggacggaggauugauUagg..................	9	1	Fm3
....uggacggaggauugauCagg..................	7	1	Fm3
....uggacggaggCuugauaagg..................	4	1	Fm3
....uggacggUggauugauaagg..................	3	1	Fm3
....uggacggaggauugauGagg..................	2	1	Fm3
....uggUcggaggauugauaagg..................	2	1	Fm3
....uggacggaggauugauaaAg..................	12	1	Fm3
....uggacggaggauugauaagg..................	3941	0	Fm3
....uggacggagAauugauaagg..................	31	1	Fm3
....uggacggaggauugCuaagg..................	1	1	Fm3
....uggacggaggauugauaaggA.................	2705	1	Fm3
....uggacggaggauugCuaaggg.................	1	1	Fm3
....uggGcggaggauugauaaggg.................	2	1	Fm3
....uggacggGggauugauaaggg.................	8	1	Fm3
....uAgacggaggauugauaaggg.................	4	1	Fm3
....Nggacggaggauugauaaggg.................	2	1	Fm3
....uggacggaggCuugauaaggg.................	5	1	Fm3
....uggacggaggauugauaaggU.................	159	1	Fm3
....uggacggaggauugauaGggg.................	19	1	Fm3
....uggacggaggauugauaaUgg.................	2	1	Fm3
....uggacggaggauugauaagCg.................	1	1	Fm3
....uggacggaggGuugauaaggg.................	16	1	Fm3
....uggacggaAgauugauaaggg.................	2	1	Fm3
....uggacggaggauugauaaggg.................	6408	0	Fm3
....uggacggCggauugauaaggg.................	1	1	Fm3
....uggacggaggauugauaagAg.................	3	1	Fm3
....uggacggaggauuCauaaggg.................	3	1	Fm3
....Aggacggaggauugauaaggg.................	1	1	Fm3
....uggacggaggauugauGaggg.................	5	1	Fm3
....Cggacggaggauugauaaggg.................	3	1	Fm3
....ugAacggaggauugauaaggg.................	1	1	Fm3
....uggacggaggauugauaaggC.................	10	1	Fm3
....uggacggaggauuAauaaggg.................	1	1	Fm3

```
                                   Star                             Mature
guguagugaagacugaagguaguucuuagcgaguccuucucuauccuucgccuaguuuauauuaaaauacuggacggaggauugauaagggcuugcggggacacgguc
......................................................................uggacggaUgauugauaaggg.................   1        1   Fm3
......................................................................uggacggaggauAgauaaggg.................   2        1   Fm3
......................................................................uggacggUggauugauaaggg.................   1        1   Fm3
......................................................................uggaAggaggauugauaaggg.................   1        1   Fm3
......................................................................uggacggagUauugauaaggg.................   1        1   Fm3
......................................................................ugUacggaggauugauaaggg.................   1        1   Fm3
......................................................................uggacgUaggauugauaaggg.................   5        1   Fm3
......................................................................uggacggaggauugauaCggg.................   18       1   Fm3
......................................................................uggacggaggaAugauaaggg.................   9        1   Fm3
......................................................................uCgacggaggauugauaaggg.................   4        1   Fm3
......................................................................uggacggaggauugauUaggg.................   10       1   Fm3
......................................................................uggacggaggauugGuaaggg.................   2        1   Fm3
......................................................................uggUcggaggauugauaaggg.................   1        1   Fm3
......................................................................uggacggagAauugauaaggg.................   50       1   Fm3
......................................................................uggaUggaggauugauaaggg.................   2        1   Fm3
......................................................................uggacggaggaCugauaaggg.................   195      1   Fm3
......................................................................uggacggaggauugauaUggg.................   29       1   Fm3
......................................................................uggacggaggauugauaaCgg.................   3        1   Fm3
......................................................................uggacggaggauugauCaggg.................   16       1   Fm3
......................................................................uggacAgaggauugauaaggg.................   1        1   Fm3
......................................................................uggacNgaggauugauaaggg.................   2        1   Fm3
......................................................................uggacggaggauugauaagUg.................   3        1   Fm3
......................................................................uggacggaggauugUuaaggg.................   5        1   Fm3
......................................................................uggacUgaggauugauaaggg.................   1        1   Fm3
......................................................................uggacgAaggauugauaaggg.................   1        1   Fm3
......................................................................uggacggaggUuugauaaggg.................   28       1   Fm3
......................................................................uggacggaggauugauaaAggc................   9        1   Fm3
......................................................................uggacggaggauugauaagggA................   5111     1   Fm3
......................................................................uggacggagUauugauaagggc................   27       1   Fm3
......................................................................uUgacggaggauugauaagggc................   54       1   Fm3
......................................................................uggacggaggauugCuaagggc................   4        1   Fm3
......................................................................ugAacggaggauugauaagggc................   41       1   Fm3
......................................................................uggacggaggaAugauaagggc................   33       1   Fm3
......................................................................uggacCgaggauugauaagggc................   9        1   Fm3
......................................................................uggacggaggauugaGaagggc................   3        1   Fm3
......................................................................uggacggaAgauugauaagggc................   10       1   Fm3
......................................................................Aggacggaggauugauaagggc................   11       1   Fm3
......................................................................uggaAggaggauugauaagggc................   33       1   Fm3
......................................................................uggacggaggauugauaaggUc................   33       1   Fm3
......................................................................uggacggCggauugauaagggc................   23       1   Fm3
......................................................................uggacggaggaCugauaagggc................   581      1   Fm3
......................................................................uggacggaggauugauaUgggc................   480      1   Fm3
......................................................................uggacggaggauugauaagggc................   149186   0   Fm3
......................................................................uggacggaggCuugauaagggc................   172      1   Fm3
......................................................................uggaUggaggauugauaagggc................   60       1   Fm3
......................................................................uggacgNaggauugauaagggc................   17       1   Fm3
......................................................................Gggacggaggauugauaagggc................   2        1   Fm3
......................................................................uCgacggaggauugauaagggc................   17       1   Fm3
......................................................................uAgacggaggauugauaagggc................   87       1   Fm3
......................................................................uggacggaUgauugauaagggc................   38       1   Fm3
......................................................................uggacggaggauugauaagCgc................   14       1   Fm3
......................................................................uggacggaggauuUauaagggc................   10       1   Fm3
......................................................................uggacggaggauugauaaggCc................   18       1   Fm3
......................................................................uggacggaggauugauaagUgc................   43       1   Fm3
......................................................................uggacggagAauugauaagggc................   189      1   Fm3
......................................................................ugUacggaggauugauaagggc................   30       1   Fm3
......................................................................uNgacggaggauugauaagggc................   1        1   Fm3
......................................................................uggacggUggauugauaagggc................   147      1   Fm3
......................................................................uggacggaggGuugauaagggc................   202      1   Fm3
......................................................................uggacAgaggauugauaagggc................   28       1   Fm3
......................................................................uggacggaggauuCauaagggc................   18       1   Fm3
......................................................................ugCacggaggauugauaagggc................   10       1   Fm3
......................................................................uggacUgaggauugauaagggc................   57       1   Fm3
......................................................................uggacggaggauuAauaagggc................   26       1   Fm3
......................................................................uggacggaggauugauaaggAc................   25       1   Fm3
......................................................................uggacggaggauugGuaagggc................   21       1   Fm3
......................................................................uggacggaggauugauCagggc................   269      1   Fm3
......................................................................uggacggaggauugUuaagggc................   20       1   Fm3
......................................................................uggacggaggauugaAaagggc................   4        1   Fm3
......................................................................uggaGggaggauugauaagggc................   6        1   Fm3
```

	Star	Mature		
guguagugaagacugaagguaguucuuagcgagucc<br>uucucuauccuucgccuaguuuauauuaaaauacuggacggaggauugauaagggcuugcggggacacgguc				
....uggGcggaggauugauaagggc................	23	1	Fm3	
....uggacggaggauugaCaagggc................	27	1	Fm3	
....Nggacggaggauugauaagggc................	74	1	Fm3	
....uggacNgaggauugauaagggc................	26	1	Fm3	
....uggacggaggUuugauaagggc................	327	1	Fm3	
....Cggacggaggauugauaagggc................	19	1	Fm3	
....uggacggaggauugauaGgggc................	493	1	Fm3	
....uggacggaggauugauaagggU................	494	1	Fm3	
....uggacggGggauugauaagggc................	59	1	Fm3	
....uggacggaggauugauGagggc................	179	1	Fm3	
....uggacggaggauugauaagggG................	18	1	Fm3	
....uggacgAaggauugauaagggc................	15	1	Fm3	
....uggacggaggauugauaagAgc................	27	1	Fm3	
....uggacggaCgauugauaagggc................	11	1	Fm3	
....uggacggagCauugauaagggc................	11	1	Fm3	
....uggacgCaggauugauaagggc................	12	1	Fm3	
....uggacggaggauugauaaCggc................	2	1	Fm3	
....uggacgUaggauugauaagggc................	76	1	Fm3	
....uggacggaggauugauaaUggc................	63	1	Fm3	
....uggUcggaggauugauaagggc................	19	1	Fm3	
....uggacggaggauGgauaagggc................	4	1	Fm3	
....uggacggaggauCgauaagggc................	27	1	Fm3	
....uggacggaggauugauUagggc................	166	1	Fm3	
....uggacggaggaGugauaagggc................	9	1	Fm3	
....ugNacggaggauugauaagggc................	1	1	Fm3	
....uggacggaggauugauaCgggc................	591	1	Fm3	
....uggCcggaggauugauaagggc................	25	1	Fm3	
....uggacggaggauAgauaagggc................	5	1	Fm3	
....uggacggaggauugauaagggcC...............	6	1	Fm3	
....uggacggaggauugaGaagggcu...............	1	1	Fm3	
....ugAacggaggauugauaagggcu...............	2	1	Fm3	
....uggacggaggUuugauaagggcu...............	8	1	Fm3	
....uggacggaggauugauaGgggcu...............	17	1	Fm3	
....uggacgUaggauugauaagggcu...............	1	1	Fm3	
....uggacgAaggauugauaagggcu...............	1	1	Fm3	
....uggacggaggauugauaagggcA...............	5357	1	Fm3	
....uggacggaggauugGuaagggcu...............	1	1	Fm3	
....uggacggGggauugauaagggcu...............	4	1	Fm3	
....uggacNgaggauugauaagggcu...............	2	1	Fm3	
....uggCcggaggauugauaagggcu...............	1	1	Fm3	
....uggacggaggauugauaaUggcu...............	3	1	Fm3	
....uggacgCaggauugauaagggcu...............	1	1	Fm3	
....uggacggaggauugauaagggcG...............	16	1	Fm3	
....ugUacggaggauugauaagggcu...............	3	1	Fm3	
....uggacggaggauugauGagggcu...............	3	1	Fm3	
....uggacggaggauugauaCgggcu...............	21	1	Fm3	
....uggacggaUgauugauaagggcu...............	1	1	Fm3	
....uggaAggaggauugauaagggcu...............	1	1	Fm3	
....uggacggaggauugauaagggUu...............	39	1	Fm3	
....uggacggaggauugauaUgggcu...............	15	1	Fm3	
....uggacggagUauugauaagggcu...............	2	1	Fm3	
....uUgacggaggauugauaagggcu...............	2	1	Fm3	
....uggacggagAauugauaagggcu...............	10	1	Fm3	
....uggacggUggauugauaagggcu...............	3	1	Fm3	
....uggacggaggauugauCagggcu...............	11	1	Fm3	
....uggacggaggaCugauaagggcu...............	20	1	Fm3	
....uggacggaggauugauaagggcu...............	4755	0	Fm3	
....Nggacggaggauugauaagggcu...............	4	1	Fm3	
....uggacggaggauugauaagggAu...............	100	1	Fm3	
....uggacggaggauugauUagggcu...............	6	1	Fm3	
....uAgacggaggauugauaagggcu...............	1	1	Fm3	
....uggacggaggCuugauaagggcu...............	7	1	Fm3	
....uggacggaggGuugauaagggcu...............	8	1	Fm3	
....uggaUggaggauugauaagggcu...............	3	1	Fm3	
....uggacggaggauugauaaAggcu...............	1	1	Fm3	
....uggacUgaggauugauaagggcu...............	5	1	Fm3	
....uggacggaggauugauaaggAcu...............	1	1	Fm3	
....uggacggaggauugauaagggAuu..............	16	1	Fm3	
....ugCacggaggauugauaagggcuu..............	1	1	Fm3	
....uggacggaggauugauaagggUuu..............	7	1	Fm3	

Star **Mature**

guguagugaagacugaagguaguucuuagcgaguccuucucuauccuucgccuaguuuauauuaaaauacuggacggaggauugauaagggcuugcggggacacgguc			
....uggacggaggauugauaaUggcuu..............	1	1	Fm3
....uggacggaggauugauaagggcuA..............	66	1	Fm3
....uggacggaggauugauaagggcuu..............	82	0	Fm3
....uggacggaggauugauaagggcAu..............	527	1	Fm3
....uggacggaggauugauaagggcuuA.............	2	1	Fm3
....uggacggaggauugauaagggcuuU.............	4	1	Fm3
....ggacggaUgauugauaag...................	1	1	Fm3
....Ugacggaggauugauaag...................	2	1	Fm3
....ggacggaggauugauaag...................	7	0	Fm3
....ggacggaggauugauaaA...................	1	1	Fm3
....ggacggagAauugauaagg..................	1	1	Fm3
....ggacggCggauugauaaggg.................	1	1	Fm3
....ggacggaggauugauaaggg.................	7	0	Fm3
....ggacggaggauugauaaggA.................	2	1	Fm3
....ggacggaggaGugauaagggc................	1	1	Fm3
....ggacggagAauugauaagggc................	3	1	Fm3
....Ugacggaggauugauaagggc................	10	1	Fm3
....ggacggaggauugauaagggc................	48	0	Fm3
....ggacggaggaCugauaagggc................	1	1	Fm3
....ggacggaggauugauaagggA................	6	1	Fm3
....ggacggaggCuugauaagggc................	1	1	Fm3
....ggacggaggauugauaagggcA...............	10	1	Fm3
....ggacggaggauugauaagggcu...............	2	0	Fm3
....ggacggaggauugauaagggAu...............	2	1	Fm3
....ggacggaggUuugauaagggcu...............	1	1	Fm3
....gacggaggauugauaagggc................	1	0	Fm3
....gacggagAauugauaagggc................	2	1	Fm3
.................................uccuucucuauccuucgccuagu..	1	0	Mm3
.................................uccuucucuauccuuUgccuagu..	1	1	Mm3
..................................ccuucucuauccuucgccG...	1	1	Mm3
..................................ccuucucuauccuucgccu...	1	0	Mm3
..................................ccuucucuauccuucgccuag...	1	0	Mm3
..................................Ucuucucuauccuucgccuag...	1	1	Mm3
..................................ccuucucuauccuuUgccuagu..	29	1	Mm3
..................................ccuucucuauccuucgccuagu..	37	0	Mm3
..................................ccuucucuauccuucgccuaguA...	1	1	Mm3
...................................cuucucuauccuucgccu...	1	0	Mm3
...................................cuucucuauccuucgccGagu..	1	1	Mm3
...................................cuucucuauccuucgccuagu..	45	0	Mm3
...................................cuucucuauccuuUgccuagu..	7	1	Mm3
...................................cuucucuauccuucgccCagu..	1	1	Mm3
...................................cuucucuauccuucgcAuagu..	1	1	Mm3
...................................cuucucuauccuucgccuaguu...	2	0	Mm3
...................................cuucucuauccuuUgccuaguuu..	1	1	Mm3
....................................uucucuauccuuUgccuagu..	1	1	Mm3
....uacuggacggaggauugauaag...................	2	0	Mm3
....uacuggacggaggauugauaagA..................	1	1	Mm3
....cuggacggaggauugauaaggg.................	1	0	Mm3
....cuggacggCggauugauaagggc................	1	1	Mm3
....cuggacggaggUuugauaagggc................	1	1	Mm3
....Auggacggaggauugauaagggc................	1	1	Mm3
....cuggacggaggauugauaagggc................	12	0	Mm3
....cuggacggaggauugUuaagggcu...............	1	1	Mm3
....uggacggaggauugaAaa....................	2	1	Mm3
....uggacggaggauugauaa....................	32	0	Mm3
....uggacggagAauugauaa....................	1	1	Mm3
....uggacggaggauugauCag...................	1	1	Mm3
....uggacggaggauugauaUg...................	4	1	Mm3
....uggacggaggauugauaaU...................	4	1	Mm3
....uggacggaggauugauaag...................	625	0	Mm3
....uggacggaggauugauGag...................	1	1	Mm3
....uggacggaggauugauaCg...................	1	1	Mm3
....uggacggaggCuugauaag...................	1	1	Mm3
....uggacggaggaCugauaag...................	9	1	Mm3
....uggaUggaggauugauaag...................	1	1	Mm3
....uggacggGggauugauaag...................	1	1	Mm3
....uggacggagAauugauaag...................	2	1	Mm3
....uggacggaggauugauaaA...................	31	1	Mm3
....uggacggaggCuugauaagg..................	1	1	Mm3

Star Mature

```
guguagugaagacugaagguaguucuuagcgaguccuucucuauccuucgccuaguuuauauuaaaauacuggacggaggauugauaagggcuugcggggacacgguc
......................................................................uggacggaggUuugauaagg..................  1      1  Mm3
......................................................................uggacggaggaCugauaagg..................  27     1  Mm3
......................................................................uggacggagAauugauaagg..................  3      1  Mm3
......................................................................uggacgUaggauugauaagg..................  1      1  Mm3
......................................................................uggacggaggauugauaGgg..................  1      1  Mm3
......................................................................uggacggaggauugauaagg..................  160    0  Mm3
......................................................................uggacggaggauugauaagU..................  5      1  Mm3
......................................................................uggacggaggauugauaagA..................  21     1  Mm3
......................................................................uggacggaggauugauaUggg.................  3      1  Mm3
......................................................................uggacggaggGuugauaaggg.................  2      1  Mm3
......................................................................uggacggagAauugauaaggg.................  7      1  Mm3
......................................................................uggacggaggauugauaGggg.................  1      1  Mm3
......................................................................uggacggaggauugauaaggA.................  91     1  Mm3
......................................................................uggacggaggUuugauaaggg.................  3      1  Mm3
......................................................................uggacggaggauugauUaggg.................  3      1  Mm3
......................................................................uggacggaggauugauaaggU.................  8      1  Mm3
......................................................................uggacggGggauugauaaggg.................  3      1  Mm3
......................................................................uAgacggaggauugauaaggg.................  1      1  Mm3
......................................................................uggacggaggauugauaCggg.................  2      1  Mm3
......................................................................uggacggaggauugauaaggg.................  590    0  Mm3
......................................................................uggacggaggauugauCaggg.................  1      1  Mm3
......................................................................uggacggaggaAugauaaggg.................  2      1  Mm3
......................................................................uggacggaggauugauGaggg.................  1      1  Mm3
......................................................................uggGcggaggauugauaaggg.................  1      1  Mm3
......................................................................uggacggaggaCugauaaggg.................  35     1  Mm3
......................................................................uggacggaggauugauaaCgg.................  1      1  Mm3
......................................................................uggacggaggauugauaaggC.................  3      1  Mm3
......................................................................uggacggaggauugauaagggA................  342    1  Mm3
......................................................................uggaGggaggauugauaagggc................  1      1  Mm3
......................................................................uggacggagAauugauaagggc................  74     1  Mm3
......................................................................uggacggagUauugauaagggc................  3      1  Mm3
......................................................................uggacggaggauugauaagggc................  23157  0  Mm3
......................................................................uggacggUggauugauaagggc................  22     1  Mm3
......................................................................uggUcggaggauugauaagggc................  4      1  Mm3
......................................................................uggGcggaggauugauaagggc................  5      1  Mm3
......................................................................uCgacggaggauugauaagggc................  1      1  Mm3
......................................................................ugAacggaggauugauaagggc................  2      1  Mm3
......................................................................uggacggaggauuUauaagggc................  4      1  Mm3
......................................................................uggacggaggauugaCaagggc................  5      1  Mm3
......................................................................Cggacggaggauugauaagggc................  4      1  Mm3
......................................................................uggacggaggauugauGagggc................  29     1  Mm3
......................................................................uggacggaUgauugauaagggc................  1      1  Mm3
......................................................................uggacggaggauugaGaagggc................  1      1  Mm3
......................................................................uAgacggaggauugauaagggc................  10     1  Mm3
......................................................................uggacNgaggauugauaagggc................  4      1  Mm3
......................................................................uggacggaggauugauaaggUc................  7      1  Mm3
......................................................................uggacggCggauugauaagggc................  3      1  Mm3
......................................................................uggacggaggauugauaaUggc................  3      1  Mm3
......................................................................uNgacggaggauugauaagggc................  1      1  Mm3
......................................................................uggacggaggauugauaaAggc................  5      1  Mm3
......................................................................uggacggaggaAugauaagggc................  9      1  Mm3
......................................................................uggacggaggaCugauaagggc................  464    1  Mm3
......................................................................uggacggaggauugauaUgggc................  76     1  Mm3
......................................................................uggacggGggauugauaagggc................  64     1  Mm3
......................................................................uggacggaggauugauaagggU................  83     1  Mm3
......................................................................uggacggaAgauugauaagggc................  1      1  Mm3
......................................................................uggacgNaggauugauaagggc................  1      1  Mm3
......................................................................uggacggaggauugUuaagggc................  3      1  Mm3
......................................................................uggacggaggauugauCagggc................  64     1  Mm3
......................................................................uggCcggaggauugauaagggc................  10     1  Mm3
......................................................................uggacggaggauugauaagAgc................  4      1  Mm3
......................................................................uggacggaggauAgauaagggc................  1      1  Mm3
......................................................................uggacggaggauugauaaggCc................  9      1  Mm3
......................................................................uggacAgaggauugauaagggc................  4      1  Mm3
......................................................................uggacgAaggauugauaagggc................  2      1  Mm3
......................................................................uggacggaggauugauaagggG................  5      1  Mm3
......................................................................ugCacggaggauugauaagggc................  1      1  Mm3
......................................................................uggacggaggGuugauaagggc................  31     1  Mm3
......................................................................uggacggaggauGgauaagggc................  2      1  Mm3
......................................................................uggacggaggauugGuaagggc................  3      1  Mm3
```

Star **Mature**

```
guguagugaagacugaagguaguucuuagcgaguccuucucuauccuucgccuaguuuauauuaaaaauacuggacggaggauugauaagggcuugcggggacacgguc
.......................................................................uggacUgaggauugauaagggc................ 5 1 Mm3
.......................................................................uggacggaggauugauaagCgc................ 2 1 Mm3
.......................................................................uggacggaggaGugauaagggc................ 1 1 Mm3
.......................................................................uUgacggaggauugauaagggc................ 6 1 Mm3
.......................................................................uggaUggaggauugauaagggc................ 6 1 Mm3
.......................................................................uggacggaggauugauaGgggc................ 104 1 Mm3
.......................................................................uggacggaggUuugauaagggc................ 72 1 Mm3
.......................................................................uggacggaggauugauaCgggc................ 91 1 Mm3
.......................................................................uggacggaggauugauUagggc................ 47 1 Mm3
.......................................................................ugUacggaggauugauaagggc................ 6 1 Mm3
.......................................................................Nggacggaggauugauaagggc................ 13 1 Mm3
.......................................................................Gggacggaggauugauaagggc................ 2 1 Mm3
.......................................................................uggacgUaggauugauaagggc................ 2 1 Mm3
.......................................................................uggacggaggCuugauaagggc................ 34 1 Mm3
.......................................................................uggacggaggauuAauaagggc................ 7 1 Mm3
.......................................................................uggaAggaggauugauaagggc................ 2 1 Mm3
.......................................................................uggacggaggauugauaaggAc................ 4 1 Mm3
.......................................................................Aggacggaggauugauaagggc................ 14 1 Mm3
.......................................................................uggacggaggauugauaagUgc................ 6 1 Mm3
.......................................................................uggacggaggauuCauaagggc................ 1 1 Mm3
.......................................................................uggacggaggauugauaaUggcu............... 1 1 Mm3
.......................................................................uggacggaggauugauaagggUu............... 4 1 Mm3
.......................................................................uggacggaggauugauaagggcu............... 1292 0 Mm3
.......................................................................uggacggaggaCugauaagggcu............... 22 1 Mm3
.......................................................................uggacggaggauugauaCgggcu............... 4 1 Mm3
.......................................................................uggacggUggauugauaagggcu............... 4 1 Mm3
.......................................................................uggacUgaggauugauaagggcu............... 1 1 Mm3
.......................................................................uggacggaggGuugauaagggcu............... 2 1 Mm3
.......................................................................uggacggaggauugauaagggcC............... 5 1 Mm3
.......................................................................uggacggGggauugauaagggcu............... 2 1 Mm3
.......................................................................uggacggaggauugaCaagggcu............... 1 1 Mm3
.......................................................................uggacggaggaAugauaagggcu............... 1 1 Mm3
.......................................................................Aggacggaggauugauaagggcu............... 1 1 Mm3
.......................................................................uggacggaggauugauaGgggcu............... 4 1 Mm3
.......................................................................uggacggaggauugauaagggAu............... 5 1 Mm3
.......................................................................uggacggaggauuUauaagggcu............... 1 1 Mm3
.......................................................................ugAacggaggauugauaagggcu............... 1 1 Mm3
.......................................................................Cggacggaggauugauaagggcu............... 1 1 Mm3
.......................................................................uggacggaggauugauaagggcG............... 27 1 Mm3
.......................................................................uggGcggaggauugauaagggcu............... 1 1 Mm3
.......................................................................uggacggaggauugauUagggcu............... 2 1 Mm3
.......................................................................uggacggagAauugauaagggcu............... 4 1 Mm3
.......................................................................uggacggaggUuugauaagggcu............... 4 1 Mm3
.......................................................................uggaAggaggauugauaagggcu............... 1 1 Mm3
.......................................................................uggacggaggauugauaagggcA............... 452 1 Mm3
.......................................................................uggacggaggauugauaagUgcu............... 1 1 Mm3
.......................................................................uggacggaggauuCauaagggcu............... 1 1 Mm3
.......................................................................uggacggaggauugauCagggcu............... 1 1 Mm3
.......................................................................uggacggaggauugUuaagggcu............... 1 1 Mm3
.......................................................................uggacggagUauugauaagggcu............... 3 1 Mm3
.......................................................................uggacggaggCuugauaagggcu............... 3 1 Mm3
.......................................................................uggacggaggauugauaUgggcu............... 2 1 Mm3
.......................................................................uggacggaggauugauaCgggcuu.............. 1 1 Mm3
.......................................................................uggacggaggauugauaagggcCu.............. 1 1 Mm3
.......................................................................uggacggaggauugauCagggcuu.............. 1 1 Mm3
.......................................................................uggacggaggauugauaagggcuu.............. 196 0 Mm3
.......................................................................uggacggaggauugauaaUggcuu.............. 1 1 Mm3
.......................................................................uggacggagAauugauaagggcuu.............. 2 1 Mm3
.......................................................................uggacggaggaCugauaagggcuu.............. 3 1 Mm3
.......................................................................uggacggaggauugauaGgggcuu.............. 1 1 Mm3
.......................................................................uggacggaggauugauaagggcuA.............. 5 1 Mm3
.......................................................................uggacggaggauugauaagggcuG.............. 3 1 Mm3
.......................................................................uggacggaggauugauaagggAuu.............. 2 1 Mm3
.......................................................................uggacggaggauugauaagggUuu.............. 6 1 Mm3
.......................................................................uggacggaggauugauaagggcAu.............. 117 1 Mm3
.......................................................................uggacggaggauugauaagggcuuA............. 1 1 Mm3
.......................................................................uggacggaggauugauaagggcuuU............. 183 1 Mm3
........................................................................ggacggaggauugauaag................... 2 0 Mm3
........................................................................ggacggaggauugauaaggg................. 2 0 Mm3
........................................................................ggacggaggauugauaaggA................. 1 1 Mm3
```

```
                                   Star                               Mature
guguagugaagacugaagguaguucuuagcgagucggcuucucuauccuucgccuaguuuauauuaaaaauacuggacggaggauugauaagggcuugcggggacacgguc
........................................................................ggacggaggaCugauaagggc................    4    1    Mm3
........................................................................ggacggaggCuugauaagggc................    1    1    Mm3
........................................................................Ugacggaggauugauaagggc................    1    1    Mm3
........................................................................ggacggagAauugauaagggc................    1    1    Mm3
........................................................................ggacggaggauugauaagggc................    7    0    Mm3
........................................................................ggacggaggauugauaagggcu...............    1    0    Mm3
.........................................................................gacggaggauugauaagggc................    1    0    Mm3
..........................................................................acggaggauugauaagggc................    2    0    Mm3
...........................................................................cggaggauugauaagggA................    1    1    Mm3
...........................................................................cggaggauugauaaggCc................    1    1    Mm3
```
